# MicroRNA-140-5p inhibits hepatocellular carcinoma by directly targeting the unique isomerase Pin1 to block multiple cancer-driving pathways

**DOI:** 10.1038/srep45915

**Published:** 2017-04-06

**Authors:** Xingxue Yan, Zhendong Zhu, Shenmin Xu, Li-nan Yang, Xin-Hua Liao, Min Zheng, Dayun Yang, Jichuang Wang, Dongmei Chen, Long Wang, Xiaolong Liu, Jingfeng Liu, Ruey-Hwa Chen, Xiao Zhen Zhou, Kun Ping Lu, Hekun Liu

**Affiliations:** 1 Fujian Key Laboratory for Translational Research in Cancer and Neurodegenerative Diseases, Institute for Translational Medicine, Fujian Medical University, Fuzhou, Fujian 350122, China; 2 Key Laboratory of Ministry of Education for Gastrointestinal Cancer, Fujian Medical University, Fuzhou, Fujian 350122, China; 3 The United Innovation of Mengchao Hepatobiliary Technology Key Laboratory of Fujian Province, Mengchao Hepatobiliary Hospital of Fujian Medical University, Fuzhou 350025, China; 4 Institute of Biological Chemistry, Academia Sinica, Taipei 115, Taiwan; 5 Division of Translational Therapeutics, Department of Medicine and Cancer Research Institute, Beth Israel Deaconess Medical Center, Harvard Medical School, Boston, MA 02215, USA

## Abstract

Hepatocellular carcinoma (HCC) is the second leading cause of cancer related-death. As a major common regulator of numerous cancer-driving pathways and a unique therapeutic target, the prolyl isomerase Pin1 is overexpressed in a majority of HCCs, whereas the mechanism underlying Pin1 overexpression remains elusive. Here we find that miR-140-5p inhibits HCC by directly targeting Pin1 to block multiple cancer-driving pathways. Bioinformatics analysis, miRNA binding and functional assays identify that miR-140-5p directly interacts with the 3′UTR of Pin1 and inhibits Pin1 translation. Furthermore, like stable Pin1 knockdown, moderate overexpression of miR-140-5p not only eliminates Pin1, but also inhibits cells growth and metastasis. Importantly, these effects of miR-140-5p are largely rescued by reconstitution of Pin1. Moreover, miR-140-5p inhibits multiple Pin1-dependent cancer pathways and suppresses tumor growth in mice. The clinical significance of these findings has been substantiated by the demonstrations that miR-140-5p is frequently down-regulated and inversely correlated with Pin1 overexpression in HCC tissues and cell lines. Given prevalent miR-140-5p downregulation in other cancers and major impact of Pin1 overexpression on activating numerous cancer-driving pathways including global miRNA downregulation, the miR-140-5p/Pin1 axis may play a major role in tumorigenesis and offer promising therapeutic targets for HCC and other cancers.

Hepatocellular carcinoma (HCC) is the second leading cause of cancer related-death, although it is the sixth most frequently diagnosed cancer of men and eleventh one of women worldwide, resulting in more than 600,000 deaths and almost as many estimated new cases each year^1^,^2^. The 5-year survival rate is still limited to 20–30% in HCC patients after surgery, mainly due to the frequent presence of metastasis, which is the fundamental feature of malignant tumors^1^,^2^,^3^. Despite much progress has been made in understanding the etiology and consequence of HCC, there is still no effective treatment available for this life-threatening disease^2^,^4^. Therefore, novel therapeutic strategies to efficiently interfere the progression of HCC patients are urgently needed^5^,^6^. In this regard, epigenetic changes in microRNAs and their target gene expression may provide new tools and opportunities for the diagnosis and treatment of HCC^7^.

Protein phosphorylation on certain Pro-directed serine or threonine residues (pSer/Thr-Pro) is a central common signaling mechanism in cell proliferation and transformation, which is regulated by many kinases and phosphatases^8^,^9^,^10^. These phosphorylated proteins uniquely adopt cis and trans conformations, a process that is catalysed by the unique peptidyl-prolyl cis/trans isomerase Pin1^11^,^12^,^13^,^14^,^15^,^16^. Pin1 specifically binds to certain Pro-directed phosphorylated serine or threonine (pSer/Thr-Pro) motifs using its WW domain, and catalyzes cis-trans isomerization of certain substrates using its PPIase domain^17^. Such pin1-catalysed conformational regulation, which can be detected by cis and trans conformation-specific antibodies^18^,^19^, has a major effect on numerous phosphorylated proteins in cancer signaling pathways^20^. It has been shown that Pin1 is overexpressed and/or overactivated in the majority of human cancers including breast, lung, colon, prostate and liver cancers, with its levels being correlated with poor outcomes in cancer patients^21^,^22^,^23^,^24^,^25^,^26^. In contrast, genetic single nucleotide polymorphism that reduces Pin1 expression is associated with reduced cancer risk for numerous cancers^27^. Moreover, Pin1 activates more than 43 oncogenes and also inactivates at least 20 tumor suppressors, leading to activation of multiple oncogenic pathways synchronously^28^. Thus, inhibiting Pin1 could obtain the unique ability to block multiple cancer-driving pathways concurrently^17^. Notably, Pin1 is overexpressed in more than 70% human HCC patients^29^,^30^ and Pin1 overexpression transforms normal liver cells^26^, where Pin1 genetic knockdown inhibits HCC tumor growth induced by HBx^31^ and multiple Pin1-dependent cancer pathways in HCC^32^. Moreover, Pin1 overexpression results in global downregulation of microRNAs (miRNAs) in HCC^33^. However, although Pin1 has been shown to be regulated by multiple mechanisms notably in breast cancer^28^,^34^,^35^, little is known so far about the molecular mechanism of Pin1 overexpression in HCC.

MiRNAs are endogenously expressed, evolutionarily conserved and small non-coding RNA molecules that have been shown to play important roles in regulating gene expression post transcriptionally by targeting the 3′UTR of mRNAs for degradation or translational repression^36^,^37^. Currently, a number of miRNAs play important roles in cancer metastasis, due to location in tumor-related genomic regions^38^. Indeed, aberrant miRNA expression has been shown to be associated with HCC, which contributes to promoting oncogenes expression or inhibiting tumor suppressors, therefore regulating multiple biological processes involved in proliferation, epithelial to mesenchymal transition (EMT) and metastasis^7^,^39^,^40^,^41^,^42^. Notably, a recent study revealed that the miR-140-5p is downregulated in HCC and affects HCC growth and metastasis by targeting FGF9 and TGFβR1 in HCC^1^. However, the cellular targets and mechanisms of miR-140-5p in the regulation of downstream signal pathways in HCC are not fully understood.

In this article, we used bioinformatics databases to identify candidate miRNAs targeting the 3′UTR of Pin1 mRNA and demonstrated that miR-140-5p inhibited Pin1 expression at the translational level in human HCC cells. Furthermore, like stable Pin1 knockdown, moderate overexpression of miR-140-5p in HCC cells not only reduced Pin1 expression, but also inhibited cells proliferation and metastasis, which were largely rescued by reconstitution of Pin1 that is resistant to miR-140-5p mediated inhibition. Importantly, such miR-140-5p overexpression also inhibited multiple Pin1-dependent cancer pathways and suppressed tumor growth of human HCC cells in mice. Moreover, miR-140-5p expression is frequently down-regulated and inversely correlated with Pin1 overexpression in human HCC tissues, as compared with those in adjacent non-cancerous liver tissues. These results together demonstrate for the first time that miR-140-5p inhibits HCC by directly targeting the unique isomerase Pin1 to block multiple cancer-driving pathways and suggest that such a new pathway could be explored for the diagnosis and treatment of HCC.

## Results

### Pin1 is a direct target for miR-140-5p in HCC

**Figure 1:**
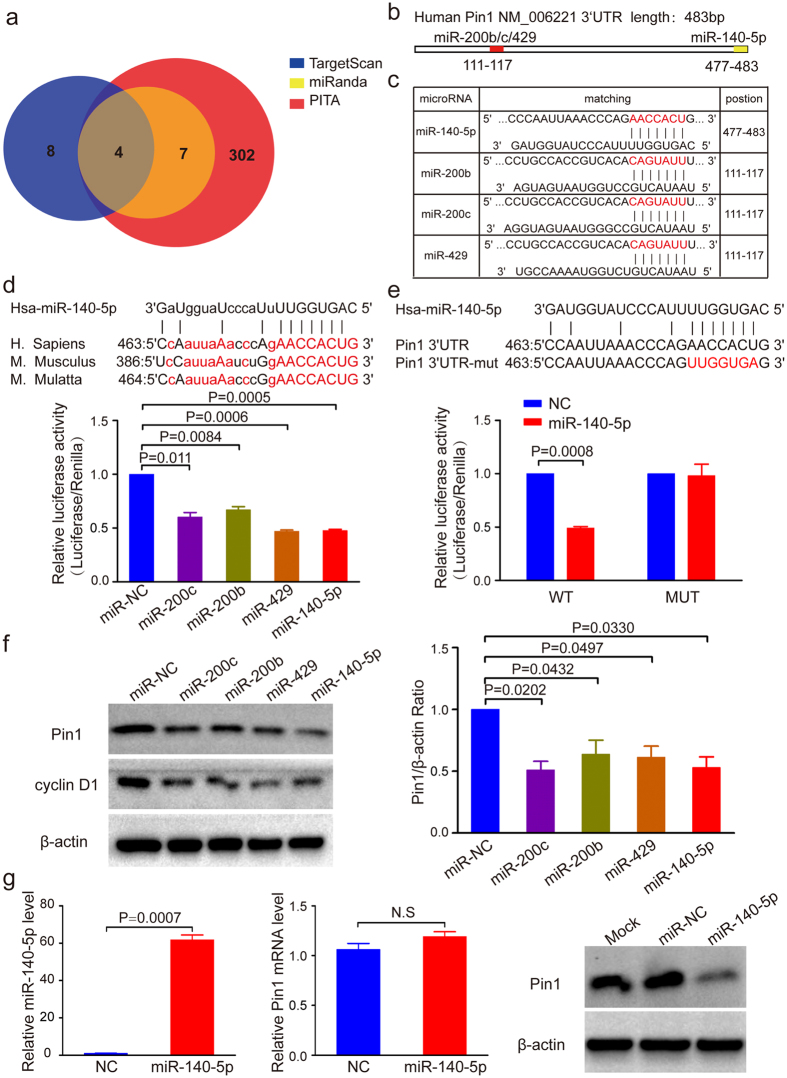
Pin1 is a direct target of miR-140-5p in HCC. (**a**) Bioinformatic prediction of candidate miRNAs targeting the Pin1 3′UTR. (**b**) Schematic diagram showing the predicted binding region of miRNAs in Pin1 3′UTR. (**c**) The predicted binding sites for miR-140-5p in Pin1 3′UTR. The red nucleotides are the seed-pairing target sites of miRNAs. (**d**) Bioinformatic analyses show Pin1 as a promising target of miR-140-5p (top panel) and miR-140-5p reduces Pin1 expression (bottom panel), as assayed by a luciferase reporter. The seed sequences of miR-140-5p within the Pin1 3′UTR are evolutionarily highly conserved across mammals as marked red. Capitalized letters are the conserved binding sites that directly interact with miR-140-5p. Dual-luciferase assay showed that miR-140-5p and miR-200s reduce luciferase activity by about 50%. (**e**) MiR-140-5p targets wild-type Pin1 3′UTR, but not its mutant. Luciferase constructs bearing a Pin1 3′UTR (WT) or Pin1 3′UTR containing mutated binding sequences of miR-140-5p (Mut) were cotransfected with miR-140-5p. Results showed that miR-140-5p reduces luciferase activity by 50%, but that was abolished when miR-140-5p binding sequences on Pin1 3′UTR was mutated. (**f**) MiR-140-5p downregulates Pin1 and cyclin D1 expression, as detected by Western blot analysis. β-actin served as loading control. (**g**) MiR-140-5p downregulates Pin1 at the translational level. Huh7 cells were infected with lentiviruses expressing miR-140-5p followed by selection with indicated concentration of puromycin. The expression of miR-140-5p and Pin1 protein were significantly increased and decreased, respectively, while Pin1 mRNA level had no significant change. In all panels, bar graphs represent mean ± SEM of three independent experiments. The statistical significance of all tests was accepted for P < 0.05.

Pin1 overexpression has been observed in ~70% human HCC patients^29^, but little is known about molecular mechanisms leading to Pin1 overexpression in HCC. To identify whether Pin1 is regulated by miRNAs, we used miRNA target prediction programs miRnada^43^, TargetScan^21^ and PITA^44^ to search for candidate miRNAs for Pin1 (Supplementary Table S1). We identified eleven candidate miRNAs with the potential targeting the 3′UTR of Pin1, which were predicted by at least two of the three bioinformatics databases. Among these eleven candidates, miR-140-5p and miR-200s (miR-200b/200c/429) were predicted by all of the three databases (Fig. 1a) and found to be frequently downregulated in HCC^1^,^45^. Interestingly, in addition to a miR-200s target site, as reported^21^,^22^, Pin1 3′UTR has a predicted miR-140-5p target site (Fig. 1b and c).

Notably, the binding site for miR-140-5p in Pin1 3′UTR is evolutionarily conserved (Fig. 1d), suggesting a potential biological significance. In order to demonstrate that miR-140-5p indeed targets Pin1, we performed a luciferase reporter assay, as described^46^. The four miRNA mimics were cotransfected with the pmirGLO vector bearing wild-type or mutated miR-140-5p binding site of Pin1 3′UTR. We observed that as shown for the positive control miR-200b and miR-200c^21^,^22^, miR-140-5p inhibited the luciferase activity of the pmirGLO plasmid with wild-type 3′UTR of Pin1 by about 50% (Fig. 1d), whereas mutation of miR-140-5p seed region in Pin1 3′UTR counteracted the regulating effects of miR-140-5p (Fig. 1e). To confirm that miR-140-5p indeed regulates Pin1 expression, we transiently transfected four different miRNA mimics into Huh7 cells for 48 hours, followed by monitoring expression of Pin1 with cyclin D1 as a functional readout for Pin1, because Pin1 controls gene transcription and protein stability of cyclin D1^20^,^47^. MiR-140-5p mimic significantly decreased both Pin1 and cyclin D1 levels (Fig. 1f), as shown for miR200b and miR200c, which have been shown to regulate Pin1 expression in other cells^21^,^22^. However, while miR-429 inhibited the luciferase activity and Pin1 protein levels as the same as miR-140-5p, miR-140-5p decreased Pin1 protein levels slightly more obvious than miR-429 (Fig. 1d and f). To further support this finding, we generated HCC cell lines Huh7 cells and PLC/PRF/5 cells stably expressing miR-140-5p using lentiviral infection. The expression of miR-140-5p, which was confirmed by RT-PCR, led to significant reduction of Pin1 protein expression without significant effects on Pin1 mRNA level (Fig. 1g), suggesting that miR-140-5p mainly regulates Pin1 at the translational level. Similarly, moderate overexpression of miR-140-5p also downregulated Pin1 expression in PLC/PRF/5 cells (Supplementary Fig. S1a). Taken together, these results support that Pin1 is a miR-140-5p downstream target.

### Moderate overexpression of miR-140-5p potently inhibits cell growth, colony formation, migration and invasion of human HCC cells

**Figure 2:**
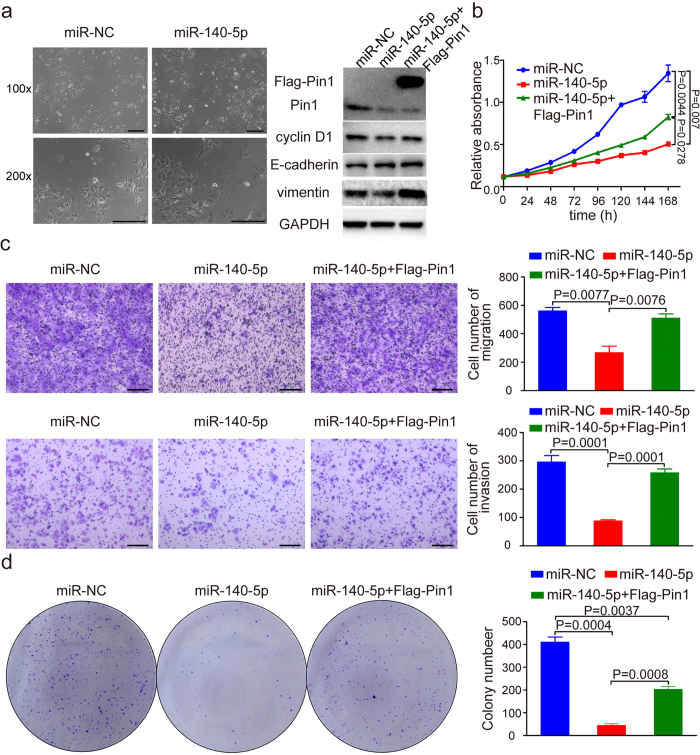
MiR-140-5p overexpression potently inhibits migration, invasion, growth and colony formation of human HCC cells *in vitro*. (**a**) Phase-contrast micrographs of indicated Huh7 cells. Scale bars, 100 μm. The protein levels of Pin1 and EMT markers in Huh7 cells after miR-140-5p overexpression were detected by Western blot assay. GAPDH served as loading control. (**b**) Cell proliferation of Huh7 cells with NC or miR-140-5p or miR-140-5p combined with expression of Flag-Pin1 resistant to miR-140-5p was detected by MTT. (**c**) Migration and invasion of Huh7 cells infected with negative control (NC) or miR-140-5p or miR-140-5p combined with expression of Flag-Pin1 resistant to miR-140-5p were assayed by transwell experiments. Scale bars, 100 μm. (**d**) The colonies of miR-140-5p overexpression cells were counted and compared with that of NC and miR-140-5p overexpression combined with overexpression of Flag-Pin1 resistant to miR-140-5p.

Pin1 overexpression in human HCC is an independent factor for poor prognosis, correlating with larger tumor size and increased portal vein invasion in HCC^48^. Given the downregulation of Pin1 by miR-140-5p in HCC cells, we wondered whether such regulation has any functional impact on HCC cells. Moderate Pin1 overexpression in non-transformed human mammary epithelial HMLE cells induces the epithelial–mesenchymal transition (EMT)^21^, which was linked to invasion and metastasis of cancer^49^. We hypothesize that miR-140-5p-mediated Pin1 downregulation might cause the mesenchymal-epithelial transition (MET) in HCCs. To test this possibility, we stably infected Huh7 HCC cells with lentiviruses expressing miR-140-5p, followed by examining its effect on cell phenotypes. MiR-140-5p overexpression not only resulted in a significant reduction in Pin1 protein levels, as compared with vector controls, but also induced epithelial-like morphological feature in Huh7 cells (Fig. 2a). To confirm that miR-140-5p-expressing cells have undergone the MET, we detected the expression of epithelial and mesenchymal markers using Western blot analysis. Indeed, miR-140-5p overexpression drastically upregulated protein levels of the epithelial marker E-cadherin, but downregulated expression of the mesenchymal markers MMP9 and vimentin (Fig. 2a and Supplementary Fig. S2a). These results are consistent with the notion that miR-140-5p overexpression causes the MET.

To further support this opinion, we carried out cell proliferation assay, colony formation assay and transwell assay for testing the migration and invasion of Huh7 cells. Strikingly, we found that miR-140-5p overexpression in Huh7 cells led to a significant decrease in the capacity of cell growth (Fig. 2b), migration and invasion (Fig. 2c), and colony formation (Fig. 2d). If these effects are specific due to miR-140-5p-mediated Pin1 downregulation, we expressed a Pin1 mutant construct that is resistant to miR-140-5p due to lack of the Pin1 3′UTR would rescue the phenotypes. Indeed, miR-140-5p-resistant Pin1 significantly rescued the expression of vimentin and cyclin D1, as a functional readout for Pin1 (Fig. 2a), as well as rescued the ability of miR-140-5p overexpression to inhibit the growth capability (Fig. 2b and d) and to reduce the migratory and invasive capacity (Fig. 2c) of Huh7 cells. To further demonstrate the function of miR-140-5p in HCC, we carried out cell proliferation assay and transwell assay in PLC/PRF/5 cells. Notably, we found that miR-140-5p overexpression in PLC/PRF/5 cells significantly reduced the expression of vimentin and cyclin D1 (Supplementary Fig. S2b), and decreased cell growth (Supplementary Fig. S2c) and migration (Supplementary Fig. S2d), which were significantly rescued by expression of miR-140-5p-resistant Pin1, consistent with the above results in Huh7 cells (Fig. 2a,b and c). The lack of a full rescue is likely due to that miR-140-5p has multiple targets such as FGF9 and TGFβR1^1^, as shown for most other miRNAs^50^. Since the original hypothesis of this study is that Pin1 is a downstream target of miR-140-5p, we would expect that Pin1 knockdown might not further enhance the tumour suppressive functions of miR-140-5p in Huh7 cells as detected by transwell migration assay. Indeed, combination of Pin1 knockdown and miR-140-5p overexpression did not significantly enhance the migratory capacity of miR-140-5p in Huh7 cells (Supplementary Fig. S2e). Taken together, these data demonstrate that miR-140-5p not only down-regulates Pin1, but also inhibits cell growth, colony formation, migration and invasion of human HCC cells *in vitro*.

### Effect of Pin1 knockdown on HCC cells

If the above functional effects of miR-140-5p on cell growth, colony formation, migration and invasion of human HCC cells is due to miR-140-5p-mediated Pin1 downregulation, we would expect that Pin1 genetic knockdown would produce the similar phenotypes. To address this possibility, we stably silenced Pin1 function by infecting Huh7 cells with lentiviruses expressing a validated shRNA of Pin1 followed by examining its effect on cell phenotypes, as described^32^. Indeed, stable knockdown of Pin1 in Huh7 cells induced the epithelial-like morphological feature (Supplementary Fig. S3a), drastically upregulated expression of the epithelial marker E-cadherin and downregulated expression of the mesenchymal marker vimentin (Supplementary Fig. S3a). Furthermore, Pin1 knockdown also significantly inhibited cell proliferation, migratory and invasive behavior of HCC cells, as detected by MTT assay (Supplementary Fig. S3b), wound healing assay (Supplementary Fig. S3c) and transwell assay (Supplementary Fig. S3d). Moreover, Pin1 knockdown also markedly inhibited cell colony formation of HCC cells (Supplementary Fig. S3e). Notably, some of these Pin1 knockdown phenotypes are similar to those observed independently as described^32^. Thus, these results indicate that both Pin1 shRNA and miR-140-5p lead to similar phenotypes, inhibiting cell growth, colony formation, migration and invasion of HCC cells *in vitro*.

### MiR-140-5P blocks multiple Pin1-dependent cancer pathways simultaneously

**Figure 3:**
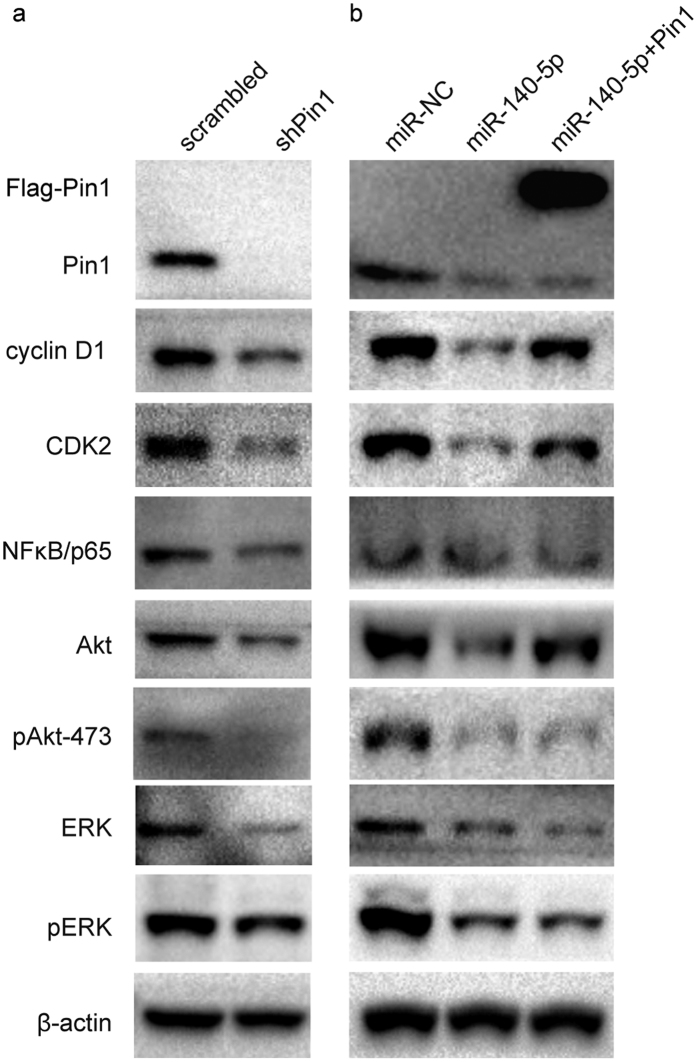
MiR-140-5p exerts potent anticancer activity against HCC by ablating Pin1 and thereby blocking multiple cancer pathways simultaneously. (**a**). Huh7 cells were infected with lentiviruses expressing scrambled or Pin1 shRNA. Cell lysates were subjected to Western blot analysis with antibodies against various proteins indicated. (**b**). Huh7 cells were infected with lentiviruses expressing miR-NC, miR-140-5p or miR-140-5p combined with overexpression of Flag-Pin1. Cell lysates were subjected to Western blot analysis with antibodies against various proteins indicated.

We and others have shown that Pin1 regulates multiple cancer pathways^28^. To further support the notion that miR-140-5p exerts potent anticancer activity against HCC by targeting Pin1, we examined the effects of miR-140-5p on a set of oncoproteins, which are substrates for Pin1 and whose protein stability is maintained by Pin1, with Pin1 knockdown as a positive control. Like Pin1 knockdown (Fig. 3a), moderate overexpression of miR-140-5p caused a significant decrease in the abundance of Pin1 and its downstream oncoproteins, including cyclin D1^51^, CDK2^52^, AKT^53^, pAKt-473^52^, ERK^28^, pERK^28^,^54^, and NF-κB p65^48^ (Fig. 3b). Moreover, these effects were partially rescued by reconstitution of miR-140-5p-resistant Pin1 (Fig. 3b). Thus, miR-140-5p exerts potent anticancer activity against HCC by ablating Pin1 and thereby blocking multiple cancer pathways simultaneously.

### MiR-140-5p inhibits HCC tumor growth by targeting Pin1 *in vivo*

**Figure 4:**
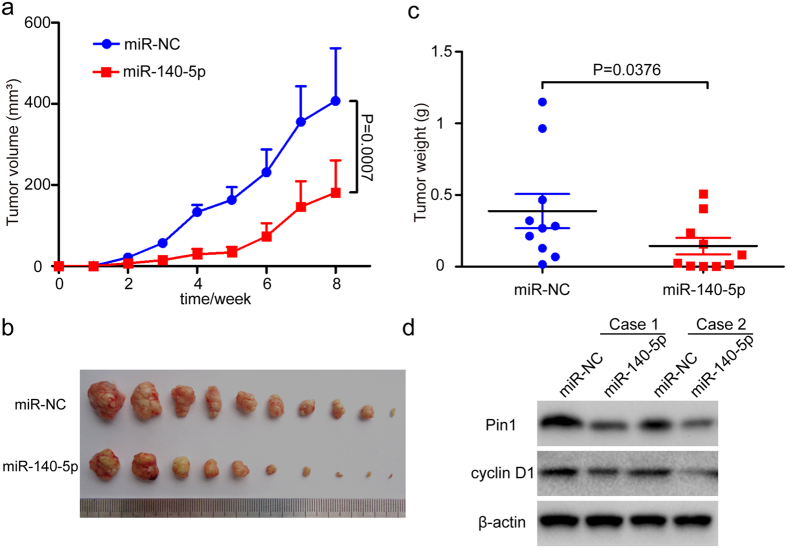
MiR-140-5p inhibits HCC tumor growth by targeting Pin1 *in vivo*. Mice were xenografted with Huh7 cells stably infected with NC or miR-140-5p lentivirus on either side of the flanks of the same mice. (**a**). Huh7 tumor volumes were measured semiweekly for 8 weeks and the curves of tumor volumes were plotted over time. (**b**). Photographic illustration of tumors harvested from nude mice at the end point (8 weeks). Each scale of the ruler represents 1 mm. (**c**). Weights of tumors in these two groups were calculated and compared. Error bar represents SEM (n = 10). (**d**). Representative immunoblots of Pin1 and cyclin D1 expression in xenograft tumors from nude mice, along with actin as a loading control.

To demonstrate that miR-140-5p could inhibit HCC tumor growth by targeting Pin1 *in vivo*, we subcutaneously injected stable miR-140-5p- or miR-NC (negative control)-expressing Huh7 cells into the either flank of the same nude mice, followed by monitoring tumor growth for 8 weeks after implantation. The size of subcutaneous tumors originated from miR-140-5p-transduced Huh7 cells was dramatically smaller than that of miR-NC (negative control)-transduced cells, as revealed by tumor growth curves, photographic illustration of final tumors or their weights (Fig. 4a,b and c). Moreover, miR-140-5p significantly decreased the abundance of Pin1 and its functional readout cyclin D1 in tumors as compared to controls (Fig. 4d), consistent with the above *in vitro* results (Figs 1f and 3c). Taken together, our data show that miR-140-5p has potent anti-tumor activity against HCC through targeting Pin1 to block multiple cancer pathways *in vitro* and *in vivo*.

### MiR-140-5p is downregulated and correlated with Pin1 overexpression in HCC cell lines and human HCC tissues

**Figure 5:**
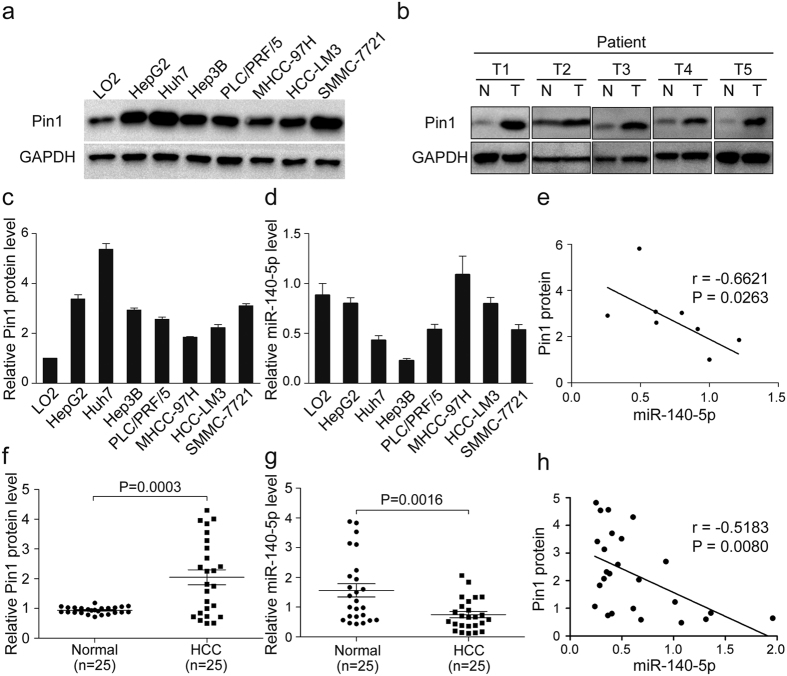
MiR-140-5p is downregulated and correlated with Pin1 overexpression in human HCC cell lines and patient specimens. (**a**) Western blotting analysis of Pin1 expression in normal human liver cell line LO2 and 7 cultured HCC cell lines. (**b**) Analysis of expression of miR-140-5p with the expression levels of Pin1 in six pairs of HCC samples (T) and adjacent non-cancerous liver tissues (N). (**c**–**e**) Relative Pin1 protein levels (normalized to GAPDH) and miR-140-5p expression levels (normalized to U6) were performed by qRT-PCR in eight liver cell lines. The expression of miR-140-5p was inversely correlated with Pin1 protein expression in HCC cell lines. (**f**–**h**) Pin1 protein and miR-140-5p levels were detected in 25 pairs of surgical specimens from HCC patients. We observed that Pin1 protein levels were significantly upregulated in HCC tissues as compared to that in ANLTs (P < 0.01), on the contrary, miR-140-5p levels were significantly downregulated in HCC tissues as compared to that in ANLTs (P < 0.01). The expression of miR-140-5p was inversely related to Pin1 expression in HCC tissues.

To demonstrate the clinical significance of miR-140-5p-mediated Pin1 regulation in HCC, we first determined expression levels of miR-140-5p and Pin1 protein by qRT-PCR and Western blot assay, respectively, in normal human liver cell line LO2 and 7 human HCC cell lines HepG2, Huh7, Hep3B, PLC/PRF/5, MHCC-97H, HCCLM3, SMMC-7721. The normal human liver cell line LO2 had lower Pin1 levels but higher miR-140-5p levels, whereas the HCC cell lines examined had higher Pin1 levels but lower miR-140-5p levels (Fig. 5a,c and d). Statistical analysis revealed Pin1 protein levels were inversely correlated with miR-140-5p levels (R = −0.6621 and P value = 0.0263) in normal and HCC cells (Fig. 5e). Next, we extended our experiments to the clinical samples obtained from HCC patients. By comparing HCC tumor tissues and paired adjacent non-cancerous liver tissues (ANLTs) of 25 HCC patients, we found that Pin1 was overexpressed in about 64% (16/25; T 1-5) of HCC tissues (Fig. 5b and f), which was consistent with the previous findings^29^. We also found that miR-140-5p was markedly reduced in HCC tissue as compared with the paired ANLTs of 25 HCC patients (Fig. 5g), as shown by a separate study^1^. More importantly, we found a significantly inverse correlation between expression levels of Pin1 and miR-140-5p (R = −0.5183 and P value = 0.0080) in human HCC samples (Fig. 5h), as expected from our above results *in vitro* and *in vivo*. These results indicate that loss of miR-140-5p is a major factor contributing to Pin1 overexpression in HCC, further supporting the significance of miR-140-5p-mediated regulation of Pin1 in HCC.

## Discussion

It has been shown that Pin1 is markedly overexpressed in a wide range of human cancers, including breast cancer^55^, prostate cancer^56^, glioblastoma^57^ and HCC^26^,^29^,^58^. Pin1 overexpression promotes tumorigenesis by activating multiple cancer-driving pathways at the same time. High Pin1 expression has been shown to be an independent factor for cancer development and poor prognosis and is along with larger tumor size and increased portal vein invasion of HCC^48^. However, while mechanisms regulating Pin1 expression have been studied in other cancers^28^, molecular mechanisms leading to Pin1 overexpression in HCC and its impact on the malignancy of HCC are still unclear. This question is important given that HCC is one of the most lethal cancers with few therapeutic options. The present study uncovers for the first time that miR-140-5p directly targets Pin1 to block multiple cancer-driving pathways and exert potent antitumor activity *in vitro* and *in vivo*.

Recently, it has been shown that deregulation of miRNAs contributes to the tumorigenesis and progression of tumor^41^,^59^,^60^, and that miRNAs carry out their function by targeting multiple genes^50^. Notably, Takata *et al*. have shown that miR-140 acts as a liver tumor suppressor by controlling NF-κB activity by directly targeting Dnmt1, and demonstrated that miR-140 is related to hepatocarcinogenesis^61^. Hao Yang *et al*. found that miR-140-5p is strikingly down-regulated in HCC and that ectopic miR-140-5p expression inhibits the capacity of HCC tumor growth and metastasis by targeting TGFβR1 and FGF9^1^.

In the present study, our bioinformatics analysis and miRNA binding and functional assays showed that miR-140-5p directly interacted with a conserved target sequence in the 3′UTR of Pin1 mRNA and inhibited Pin1 translation in human HCC cells. Furthermore, like Pin1 knockdown, miR-140-5p exerted potent anticancer activity against HCC by targeting Pin1 to block multiple Pin1-dependent cancer pathways simultaneously, thereby suppressing the migratory, invasive and proliferous capacity of HCC cells. Importantly, these effects of miR-140-5p were significantly, but not fully rescued by reconstitution of Pin1 resistant to miR-140-5p. These results are consistent with the previous findings that miR-140-5p has other targets such as TGFβR1 and FGF9^1^. Interestingly, some downstream targets of TGFβR1 and FGF9 such as TGF-β and the ERK/MAPK signaling pathways^62^,^63^ are regulated by Pin1^28^. For example, in our study, miR-140-5p suppressed the expression of a few endogenous ERK/MAPK pathway-related proteins (such as ERK and pERK) (Fig. 4b) by ablating Pin1 and thereby inhibited the activity of ERK/MAPK signaling pathway. Therefore, Pin1 is likely important for miR-140-5p to affect HCC growth and metastasis by targeting FGF9 and TGFβR1. Importantly, we found that miR-140-5p also inhibits many other Pin1 substrate oncoproteins. Moreover, miR-140-5p decreased tumor growth of HCC by ablating Pin1 and its downstream target cyclin D1 *in vivo*. Finally, expression of miR-140-5p was markedly reduced and correlated with Pin1 overexpression in human HCC tissues and cell lines compared to their respective normal controls. These results together indicate that loss of miR-140-5p contributes to tumor growth and metastasis of HCC, at least in part, through the up-regulation of Pin1 and its multiple cancer-driving pathways simultaneously.

Interestingly, in addition to HCC^1^, miR-140-5p has been shown to be gradually lost along with tumor progression and related to metastatic disease in ovarian cancer^64^, colorectal cancer^65^, lung cancer^66^, tongue cancer^67^. Notably, Pin1 has been shown to overexpress in most human cancer and activate at least 43 oncoproteins and inactivate at least 20 tumor suppressors^28^. Our results suggest that the down-regulation of miR-140-5p might also promote tumor growth and metastasis of many other cancers by directly increasing Pin1 expression to activate multiple cancer-driving pathways. Notably, Pin1 overexpression has been shown to reduce pre-miRNA export from the nucleus, resulting in global downregulation of miRNAs in liver cancer^33^. Therefore, the deregulation of the miR-140-5p/Pin1 interaction may represent an aggressive molecular lesion in cancer development by resulting in global reduction of miRNA and activation of numerous cancer-driving pathways, offering novel therapeutic targets not only for HCC, but also for other cancers.

## Materials and Methods

### Cell cultures

The normal human liver cell line LO2, and HCC cell lines HepG2, Huh7, Hep3B, PLC/PRF/5, MHCC-97H, HCCLM3, SMMC-7721 were purchased from the Type Culture Collection of the Chinese Academy of Sciences, Shanghai, China. LO2, Huh7, MHCC-97H, HCCLM3 were cultured in DMEM (Invitrogen), supplemented with 1% antibiotics (GIBCO) and 10% fetal bovine serum (GEMINI). The HepG2, Hep3B, PLC/PRF/5 cell lines were cultured in MEM (Invitrogen) supplemented with 10% FBS and 1% antibiotics. The SMMC-7721 was cultured in RPMI1640 (Invitrogen) with 1% Penicillin-Streptomycin and 10% FBS. All cells were cultured in 5% CO_2_ humid atmosphere at 37 °C.

### Clinical specimens

All studies involving human subjects were approved by the Institutional Review Board at Mengchao Hepatobiliary Hospital of Fujian Medical University, and performed in accordance with the relevant guidelines and regulations. Informed consent was obtained from all patients at Mengchao Hepatobiliary Hospital of Fujian Medical University before we carried out our studies. Matched fresh HCC tissues and adjacent non-cancerous liver tissues (ANLTs) of 25 HCC patients were collected at the time of hepatic resection and immediately frozen in liquid nitrogen and then stored at −80 °C for RNA and Protein isolation^1^. All HCC tissues were further affirmed by pathological examination. The clinicopathological characteristics of these fresh samples were provided in the Supplementary Table S2.

### Oligonucleotides and Plasmids

MiRNA-140-5p, miRNA-200b, miRNA-200c, miRNA-429 and NC mimics were synthesized by Genepharma (Shanghai, China). The oligonucleotides of these miRNA mimics are shown in the Supplementary Table S3.

The 3′-UTR of Pin1 was amplified by PCR using the primers pmirGLO-Pin1-3′-UTR-WT (wild-type)-Reverse and pmirGLO-Pin1-3′-UTR-WT-Forward and then cloned in multiple cloning site (MCS), downstream of the luciferase gene, of the pmirGLO Dual-Luciferase miRNA Target Expression Vector (promega). This plasmid was sequenced and named pmirGLO-Pin1-3′-UTR-WT. Procedure for pmirGLO-Pin1-3′-UTR-Mut (mutant), which carried a replacement of seven nucleotides in the binding site, was performed by using the primers pmirGLO-Pin1-3′-UTR-Mut-Reverse and pmirGLO-Pin1-3′-UTR-Mut-Forward as described above. The oligonucleotides of these primers are shown in the Supplementary Table S3.

The lentiviral plasmid for expression of miR-140-5p and NC (Negative control) were LV-3 (pGLVH1/GFP + Puro) purchased from Genepharma. For overexpression of Pin1, the CDS were subcloned into the pBybe lentiviral vector, and for stable knockdown of Pin1, the shRNA construct was subcloned into the pLKO.1 lentiviral vector, as described previously^21^. The production of lentiviruses as well as the infection of target cells was on the basis of previous description^68^. The cells were selected using puromycin after infection. Cell lines were infected before each group of experiments and each experiment was performed independently at least three times.

### Transient Transfection

The miRNA mimics (50 nM) were transfected into cells using TurboFect Transfection Reagent (Thermo Scientific) according to the directions of manufacturer. The cells (5 × 10^4^) were plated in each well of the 24-well plate and incubated overnight. Mimics and TurboFect were mixed in Opti-MEM, coincubated for 20 min, and then added to the 24-well plate. Cells were cultured with the mixture in medium and harvested at an indicated time point for specific experiments.

### Luciferase Reporter Assays

For the reporter assays, HEK293T cells (5 × 10^4^) were seeded in 24-well plate with 30% confluence, twelve hours later, the cells were cotransfected with 50 ng pmirGLO-Pin1-3′-UTR luciferase plasmid and 50 nM miRNA mimics using TurboFect Transfection Reagent (Thermo Scientific). After 48 hours of transfection, the HEK293T cells were harvested for detection using the Dual-Luciferase Reporter Assay system (Promega) according to the specifications of the manufacturer. The Renilla luciferase activities were used to normalize the transfection efficiency.

### Western blot analysis

Total proteins were extracted by placing cell lines and HCC specimens in lysis buffer at 4 °C for 30 min. The protein samples were separated by using SDS-polyacrylamide gel electrophoresis (PAGE) and then transferred onto PVDF membrane. The PVDF membranes were incubated with primary antibodies, anti-Pin1 (home made), anti-MMP9 (1:200; D261999; Sangon Biotech), anti-E-cadherin (1:500; #3195 S; Cell Signaling Technology), anti-vimentin (1:1,000; #3932 S; Cell Signaling Technology), anti-cyclinD1 (1:500; #2978 S; Cell Signaling Technology), anti-CDK2 (1:500; #2546 S; Cell Signaling Technology), anti-Akt (1:1,000; #9272 S; Cell Signaling Technology), anti-p-Akt(Ser 473) (1:500; #9271 S; Cell Signaling Technology), anti-ERK (1:500; 13-6200; Invitrogen), anti-pERK (1:1,000; #9101 S; Cell Signaling Technology), anti-NF-κB p65 (1:1,000; #8242 S; Cell Signaling technology) and then probed with a secondary antibody (1:5,000; Merck Millipore). β-actin (1:8,000; #HC201-02; TransGen Biotech) or GAPDH (1:8,000: #HC301-02; TransGen Biotech) was used as a protein loading control.

### Total RNA Isolation, RT-PCR, and Quantitative Real-time PCR

Total RNA was extracted from HCC specimens and cell lines using RNAiso Plus reagent (TaKaRa). Complementary DNA (cDNA) was reverse transcribed from the total RNA using PrimeScript™ RT reagent Kit with gDNA Eraser (TaKaRa). Quantitative Real-time PCR was performed using SYBR Premix Ex Taq (TaKaRa) according to the manufacturer’s protocol. The primer sequences used in this study are provided in Supplementary Table S3.

Total RNA containing miRNA was isolated from HCC specimens and cell lines using mirVana™ miRNA Isolation Kit (Invitrogen), and the reverse transcription reaction was performed using TaqMan^®^ MicroRNA Reverse Transcription Kit (Invitrogen). Quantification of miR-140-5p was performed using TaqMan^®^ MicroRNA Assays (Invitrogen) in a reaction containing 10 ng of total cDNA following the manufacturer’s protocol and the specific primers for miR-140-5p was designed by Applied Biosystems. The relative expression of miRNA-140-5p in tumor tissues against ANLTs and among different cell lines was obtained by comparing the cycle threshold (Ct) values which were normalized by the internal control RNU6B (U6 snRNA). Each sample was detected in triplicates.

### Cell Proliferation, Cell Cycle, Cell Colony Formation assays

Cell proliferation was determined by counting the number of cells or using 3-(4, 5-dimethylthiazol-2-yl)-2, 5- diphenyltetrazolium bromide (MTT) assays. For cell colony formation assays, Huh7 cells (4 × 10^3^) were seeded in each well of 6-well plates and cultured for 2 weeks at 5% CO_2_ 37 °C. The numbers of colonies per well were counted after staining with crystal violet. All studies were conducted with 3 replicates.

### *In Vitro* Wound Healing, Migration and Invasion Assays

Cells (4 × 10^5^) were seeded into 12-well plate coated with Collagen. Until the cells reached 95% confluence, wound healing assays were carried out with a sterile 10 μL pipette tip to straightly scratch 2 lines through the confluent monolayer. The cells were cultured with fresh medium for another 48 h, and then photographed for calculating wound closure. The percent of wound closure was calculated using Image J software.

For migration assay, cells (5 × 10^4^) in serum-free medium were seeded into the upper chamber without matrigel (Merck Millipore), and 48 hours later, the cells in the upper chamber were removed. The number of cells that adhering to the lower membrane was counted after staining with 0.1% crystal violet. For the invasion assay, cells (5 × 10^4^) in serum-free medium were seeded into the upper chamber of the insert with matrigel (BD Biosciences), and 48 hours later, the cells in the upper chamber were removed. The number of cells that adhering to the lower membrane was counted after staining with 0.1% crystal violet. All studies were conducted with 3 replicates.

### HCC Mouse Model

The hepatocellular carcinoma nude mice model was constructed as previously described^69^. Briefly, cells (3 × 10^6^) were subcutaneously injected into the bilateral upper flank regions of the nude mice (3-4 weeks of age, female, BALB/c). Two month later, the mice were sacrificed when their tumor volume reached 1000 mm^3^, and the subcutaneous tumors were removed for calculating the size as follows: tumor volume (mm3) = (L × W^2^)/2, where L = long axis and W = short axis^1^. BALB/c nude mice were housed in laminar flow cabinets with free access to food and water in Laboratory Animal Center of Fujian Medical University. All of animal experiments were performed in accordance with the animal protocols and regulations approved by FJMU Experimental Animal Ethics Committee of Fujian Medical University.

### Statistical Analysis

Statistical analysis was performed using SPSS (v. 22.0, Chicago, IL). All data are presented as the mean ± SEM, and then two-tailed Student’s t test or analysis of variance test (ANOVA) was used to compare unpaired samples. Paired Student’s t test was used to compare paired groups of samples. The statistical significance of all tests was accepted for P < 0.05, P < 0.01 and P < 0.001.

## Additional Information

**How to cite this article:** Yan, X. *et al*. MicroRNA-140-5p inhibits hepatocellular carcinoma by directly targeting the unique isomerase Pin1 to block multiple cancer-driving pathways. *Sci. Rep.* **7**, 45915; doi: 10.1038/srep45915 (2017).

**Publisher's note:** Springer Nature remains neutral with regard to jurisdictional claims in published maps and institutional affiliations.

## Supplementary Material

Supplementary Information
