# Supplementary material for: MicroRNA-140-5p inhibits hepatocellular carcinoma by directly targeting the unique isomerase Pin1 to block multiple cancer-driving pathways
Source: Sci Rep. 2017 Apr 6;7:45915. doi: 10.1038/srep45915 (PMC5382892; doi:10.1038/srep45915)
Supplement: Supplementary Information [file srep45915-s1.pdf]

Title：

MicroRNA-140-5p inhibits hepatocellular carcinoma by directly targeting the unique isomerase Pin1 to block multiple cancer-driving pathways

Xingxue Yan [1,2,*], Zhendong Zhu [1,2*], Shenmin Xu [1,2*], Li-nan Yang [1,2], Xin-Hua Liao [1,2], Min Zheng [1,2], Dayun Yang [1,2], Jichuang Wang [1,2], Dongmei Chen [1,2], Long Wang [1,2], Jingfeng Liu [3], Xiaolong Liu [3], Ruey-Hwa Chen [4], Xiao Zhen Zhou [1,2,5], Kun Ping Lu [1,2,5,#] and Hekun Liu [1,2,#]

[1]Fujian Key Laboratory for Translational Research in Cancer and Neurodegenerative Diseases, Institute for Translational Medicine, Fujian Medical University, Fuzhou, Fujian 350122, China.

[2]Key Laboratory of Ministry of Education for Gastrointestinal Cancer, Fujian Medical University, Fuzhou, Fujian 350122, China.

[3]The United Innovation of Mengchao Hepatobiliary Technology Key Laboratory of Fujian Province, Mengchao Hepatobiliary Hospital of Fujian Medical University, Fuzhou 350025, China.

[4]Institute of Biological Chemistry, Academia Sinica, Taipei 115, Taiwan.

[5]Division of Translational Therapeutics, Department of Medicine and Cancer Research Institute, Beth Israel Deaconess Medical Center, Harvard Medical School, Boston, MA 02215, USA

*These authors contribute equally to the work.

# Correspondence should be addressed to Hekun Liu at fjlhk@163.com or Kun Ping Lu at klu@bidmc.harvard.edu

Key Words: miR-140-5p; Pin1; hepatocellular carcinoma; metastasis

Supplementary Tables.

Table S1. MiRNA candidates potentially targeting the 3'-untranslated region of Pin1 mRNA were predicted by at least two of the five bioinformatics databases

Predicted miRNAs / Predicted databases	miRnada	PITA	TargetScan
has-miR-140		√	√
has-miR-140-5p	√	√	√
has-miR-200b	√	√	√
has-miR-200c	√	√	√
has-miR-429	√	√	√
has-miR-488	√	√	
has-miR-450a	√	√	
has-miR-370	√	√	
has-miR-548a		√	√
has-miR-876-3p		√	√
has-miR-1244		√	√

Table S2. Clinicopathological characteristics of studied patients and expression of Pin1 and miR-140-5p in HCC

Factor	NO.	(%)
**Gender**		
Male	22	88
Female	3	12
**Age (years)**		
≤60	16	64
>60	9	36
**Cirrhosis**		
Presence	20	80
Absence	5	20
**Tumor size (cm)**		
≤5	9	36
>5	16	64
**Tumor nodule number**		
Solitary	18	72
Multiple (≥2)	7	28
**Capsular formation**		
Presence	15	60
Absence	10	40
**Edmondson-Steiner grade**		
I-II	6	24
III-IV	19	76
**Vein invasion**		
Presence	18	72
Absence	7	28
**TNM stage**		
Stage I	3	12
Stage II	8	32
Stage III	14	56
**HBV**		
Negative	2	8
Positive	23	92
**Pin1 expression**		
Low expression	9	36
High expression	16	64
**MiR-140-5p expression**		
Low expression	17	68
High expression	8	32

Table S3. Sequence of oligonucleotides for plasmid construct, siRNA, qRT-PCR and miRNAs oligonucleotide

Assay		Sequence 5' →3’
**PCR**		
Pin1	F	AAGATGGCGGACGAGGAG
	R	CACTCAGTGCGGAGGATGAT
GAPDH	F	ACCCACTCCTCCACCTTTG
	R	CTCTTGTGCTCTTGCTGGG
**Plasmid construction**		
pmirGLO-Pin1-3’UTR-WT	F	CTAGTTGTTTAAACGAGCTCGGGTGGGGAGCCCAGGCCTG
	R	ATGCCTGCAGGTCGACGCAGAGCAGTGGTTCTGGGT
pmirGLO-Pin1-3’UTR-Mut	F	CTAGTTGTTTAAACGAGCTCGGGTGGGGAGCCCAGGCCTG
	R	ATGCCTGCAGGTCGACGCAGAGCTCACCAACTGGGT
**miRNAs oligonucleotide**		
miRNA-200b mimics		UAAUACUGCCUGGUAAUGAUGA
		AUCAUUACCAGGCAGUAUUAUU
miRNA-200c mimics		UAAUACUGCCGGGUAAUGAUGGA
		CAUCAUUACCCGGCAGUAUUAUU
miRNA-429 mimics		UAAUACUGUCUGGUAAAACCGU
		GGUUUUACCAGACAGUAUUAUU
miRNA-140-5p mimics		CAGUGGUUUUACCCUAUGGUAG
		ACCAUAGGGUAAAACCACUGUU
Negative control		UUCUCCGAACGUGUCACGUTT
		ACGUGACACGUUCGGAGAATT

Supplementary Figures.

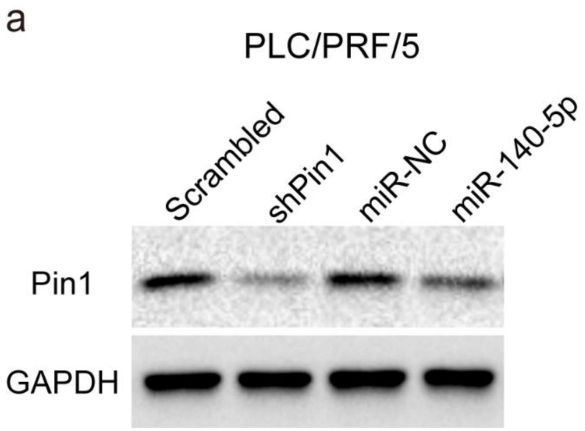

Figure S1. Effect of miR-140-5p overexpression on the protein levels of Pin1 in PLC/PRF/5 cells.

(a). Like stable Pin1 knockdown, moderate overexpression of miR-140-5p downregulates Pin1 expression in PLC/PRF/5 cells, as detected by immunoblotting analysis. GAPDH served as loading control.

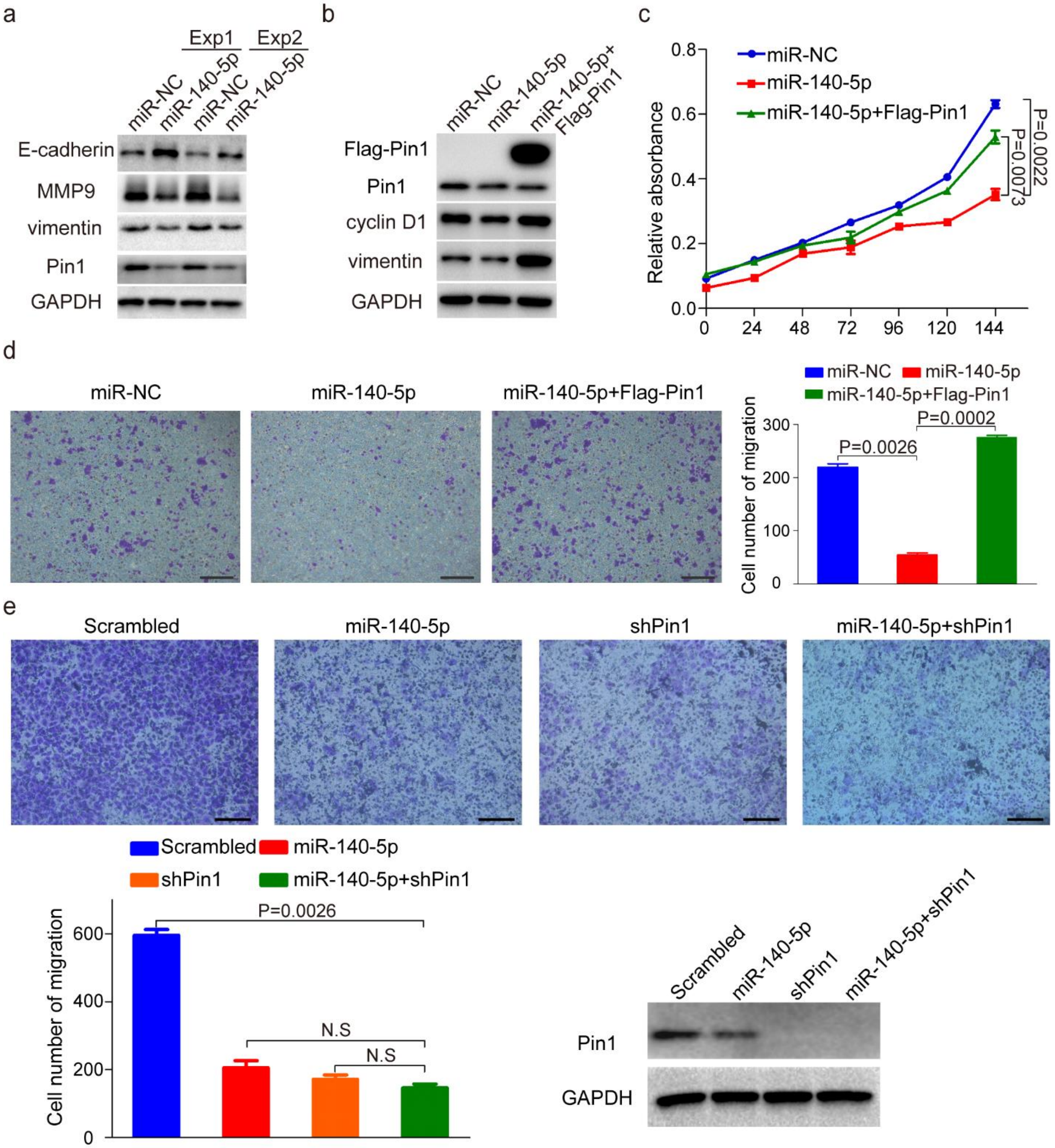

Figure S2. Effect of Pin1 overexpression or stable Pin1 knockdown on the function of miR-140-5p.

(a). The protein levels of Pin1 and EMT markers in Huh7 cells after miR-140-5p overexpression were detected by Western blot assay. GAPDH served as loading control.

(b). The protein levels of Pin1, cyclinD1 and vimentin in PLC/PRF/5 cells after co-transfected with miR-140-5p and Flag-Pin1 were detected by Western blot assay. GAPDH served as loading control.

(c). Cell proliferation of PLC/PRF/5 cells with NC, miR-140-5p or miR-140-5p combined with expression of Flag-Pin1 resistant to miR-140-5p was detected by MTT.

(d). Migration of PLC/PRF/5 cells infected with NC, miR-140-5p or miR-140-5p combined with expression of Flag-Pin1 resistant to miR-140-5p were assayed by transwell experiments. Scale bars, 100 μm.

(e). Migration of Huh7 cells infected with Scrambled, miR-140-5p, shPin1 or shPin1 combined with expression of miR-140-5p were assayed by transwell experiments. Scale bars, 100 μm. The protein levels of Pin1 in Huh7 cells were detected by Western blot assay. GAPDH served as loading control.

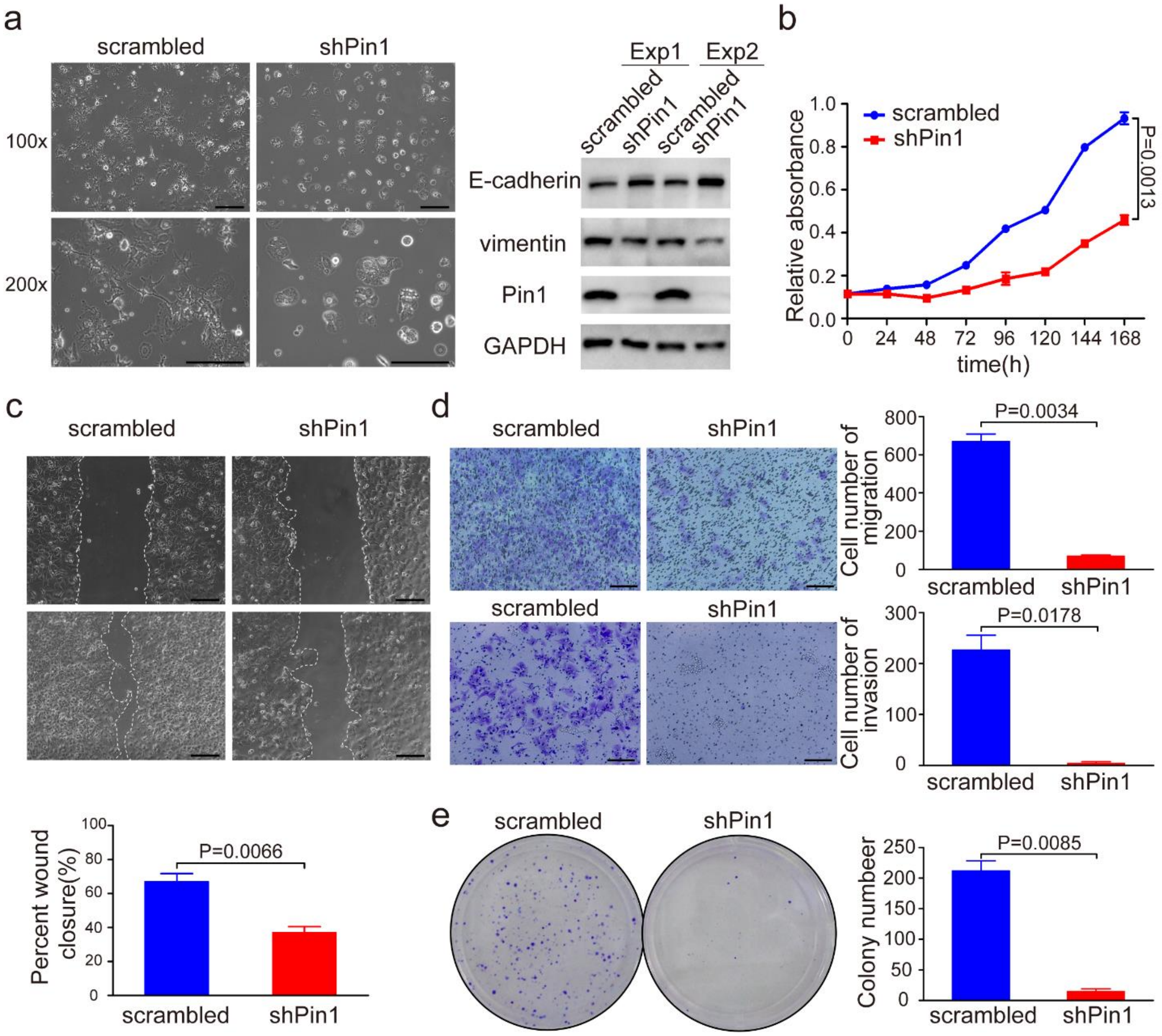

Figure S3. Stable knockdown of Pin1 inhibits migration, invasion, growth and colony formation of human HCC cells in Huh7.

(a). Phase-contrast micrographs of indicated Huh7 cells. Scale bars, 100 μm. The protein levels of EMT markers and Pin1 in two indicated Huh7 cells. GAPDH served as loading control.

(b). The growth of indicated Huh7 cells was performed by using MTT assay.

(c,d). The wound-healing assay (c) and transwell assay (d) of Huh7 cells infected with lentivirus. The invasion assay was measured with Matrigel-coated transwell experiments. Scale bars, 100 μm.

(e). The colonies of indicated Huh7 cells were counted and compared with that of scrambled.

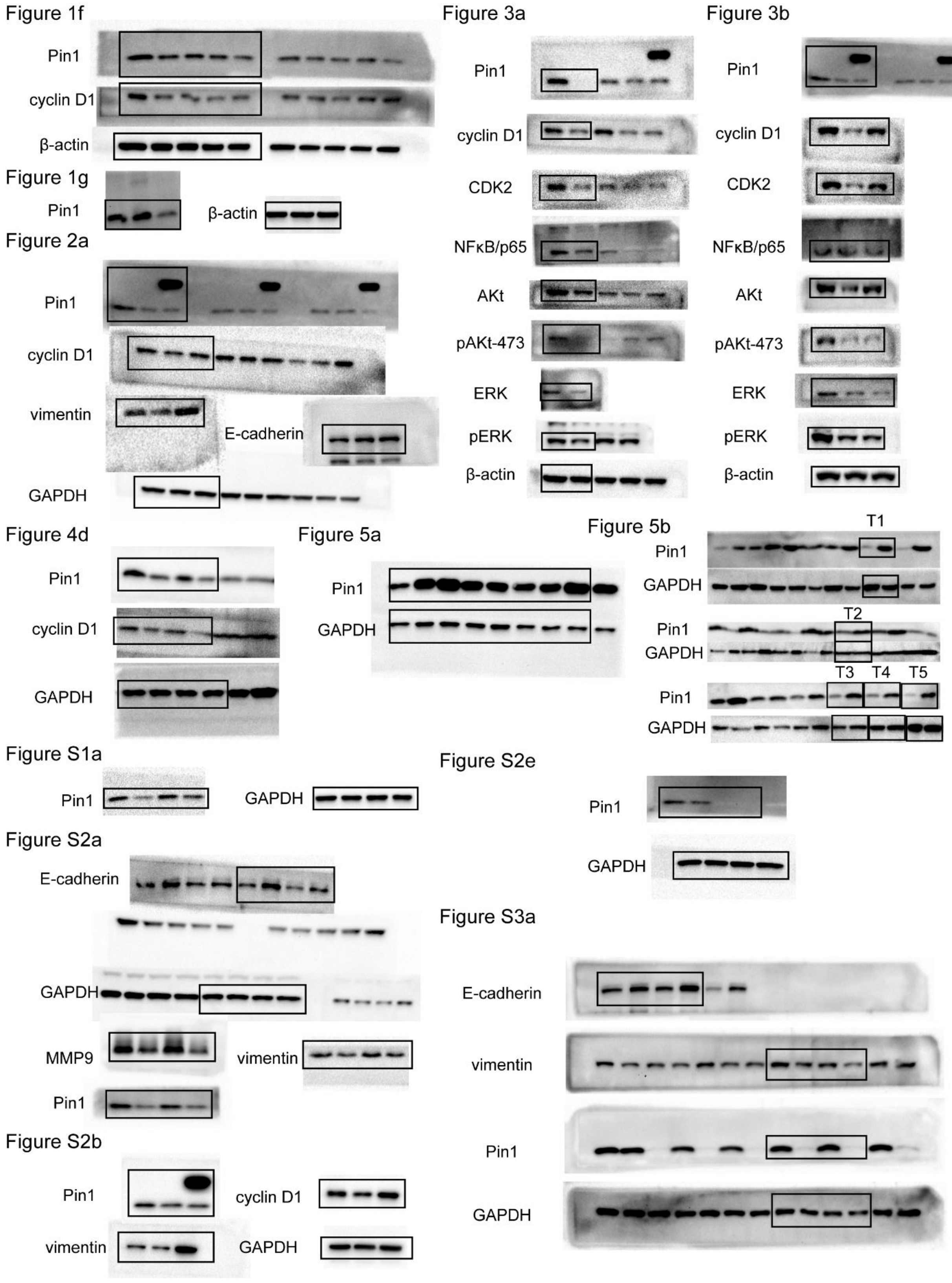

Figure S4. Uncropped images for the key experiments in the main figure.
